# Establishment of a Pretreatment Nomogram to Predict the 6-Month Mortality Rate of Patients with Advanced Biliary Tract Cancers Undergoing Gemcitabine-Based Chemotherapy

**DOI:** 10.3390/cancers13133139

**Published:** 2021-06-23

**Authors:** Chiao-En Wu, Wen-Kuan Huang, Wen-Chi Chou, Chia-Hsun Hsieh, John Wen-Cheng Chang, Cheng-Yu Lin, Chun-Nan Yeh, Jen-Shi Chen

**Affiliations:** 1 Department of Internal Medicine, Division of Haematology-Oncology, Chang Gung Memorial Hospital at Linkou, College of Medicine, Chang Gung University, 333 Taoyuan, Taiwan; 8805017@adm.cgmh.org.tw (C.-E.W.); medfoxtaiwan@gmail.com (W.-K.H.); f12986@cgmh.org.tw (W.-C.C.); wisdom5000@cgmh.org.tw (C.-H.H.); wen1902@cgmh.org.tw (J.W.-C.C.); 2 Department of Gastroenterology, Chang Gung Memorial Hospital at Linkou, College of Medicine, Chang Gung University, 333 Taoyuan, Taiwan; 8805035@cgmh.org.tw; 3 Department of General Surgery, Chang Gung Memorial Hospital at Linkou, College of Medicine, Chang Gung University, 333 Taoyuan, Taiwan

**Keywords:** biliary tract cancer, chemotherapy, gemcitabine, prognostic factors, nomogram

## Abstract

**Simple Summary:**

The development of a simple tool that uses pretreatment clinical factors to predict the 6-month mortality rate of patients with advanced biliary tract cancer is critical in order to assist physicians in evaluating treatment options and outcomes. We established a nomogram including four independent pretreatment factors—gender, monocyte to lymphocyte ratio, alkaline phosphatase, and liver metastasis—based on data from 202 patients undergoing gemcitabine-based chemotherapy. The performance of this nomogram for 6-month mortality-risk prediction was promising and feasible, providing clinicians and patients with additional information for evaluating therapeutic options.

**Abstract:**

Background: The estimation of mortality risk among patients diagnosed with advanced cancer provides important information for clinicians and patients in clinical practice. Currently, gemcitabine-based chemotherapy regimens are the standard treatment for patients with advanced biliary tract cancer (BTC). We aimed to develop a nomogram to predict the 6-month mortality rate among patients with advanced BTC to help physicians evaluate treatment options and outcomes. Patients: We conducted a retrospective analysis to evaluate the 6-month mortality rate among patients with advanced BTC who underwent gemcitabine-based chemotherapy from 2012 to 2018. Data regarding pretreatment factors and the clinical response to treatment were collected. Univariate and multivariate analyses were performed to identify independent factors for nomogram creation. Results: A total of 202 advanced BTC patients who were treated with gemcitabine-based chemotherapy were included in this analysis. No difference in survival was identified between patients undergoing gemcitabine monotherapy and those treated with gemcitabine combined with other cytotoxic agents. The univariate analysis revealed 10 significant factors, while the multivariate analysis identified four independent factors, including gender, monocyte to lymphocyte ratio (MLR), alkaline phosphatase (ALP), and liver metastasis, which were used to establish the nomogram. The performance of this nomogram for the prediction of 6-month mortality risk was found to be promising and feasible based on logistic regression. Conclusion: A nomogram based on four independent pretreatment factors, including gender, MLR, ALP, and liver metastasis, was established to predict the 6-month mortality risk in patients with advanced BTC; it can provide clinicians and patients with additional information when evaluating treatment outcomes.

## 1. Introduction

Biliary tract cancers (BTCs) represent a group of malignancies that arise from the epithelium of the biliary tract;the reported incidence of BTC has been increasing worldwide [1,2,3]. Most BTCs, including intrahepatic cholangiocarcinoma (ICCA), common bile duct cancer, gallbladder cancer, and ampullary cancer, have aggressive biological behaviors and are diagnosed at an advanced stage, resulting in a poor prognosis [4,5].Risk factors and molecular alterations differ among BTC subtypes [5].

Chemotherapy with gemcitabine plus cisplatin (GC) has been the standard first-line chemotherapy option since the ABC-02 trial was published in 2010 [6]. An alternative option, gemcitabine plus TS-1 (GS), was found to be non-inferior to GC in the Japanese Phase III FUGA-BT trial [7]. Another regimen, gemcitabine plus oxaliplatin (GEMOX), has been used as the control arm in Phase II and III trials for BTCs [8,9,10]. Based on these outcomes, gemcitabine-based therapy pairings are widely used to treat patients with advanced BTC. Several clinical trials have evaluated targeted molecular therapies combined with chemotherapy; however, none of the Phase III trials demonstrated significant improvements in either progression-free survival (PFS) or overall survival (OS) [11,12,13].

We previously reported the efficacy and safety of the GC chemotherapy regimen in 30 patients with advanced BTC in a study published in 2012 [14], which demonstrated that this regimen was feasible with a manageable toxicity in clinical practice. In a recent study published in 2020, we identified possible predictive and prognostic factors in 118 patients treated with GC [15]. However, in the real-world cohort the median survival reported for the clinical treatment of BTC with the GC regimen was only 8.4 months, which was shorter than the survival rates reported by clinical trials (OS = 11.7 months in ABC-02 trial) [6], possibly due to the inclusion of ineligible participants pertaining to patient comorbidities, performance status, or extremely aggressive and fast-growing tumors. These findings suggest that an estimation of the 6-month mortality risk for patients with advanced BTC that is based on real-world data, rather than data reported in clinical trials, might provide important information for clinicians and patients when deciding treatment strategies. In contrast, it has been reported that the accuracy of clinicians’ predictions varies widely and they consistently overestimate survival [16]. Therefore, the establishment of an objective nomogram based on real-world experience is warranted. Gemcitabine-based chemotherapy regimens remain the primary first-line treatment option in clinical practice outside of clinical trials; therefore, we aimed to develop a nomogram capable of predicting the 6-month mortality rate among Taiwanese patients with advanced BTC to guide physicians in the evaluation of treatment options and outcomes.

## 2. Materials and Methods

### 2.1. Patients and Data Collection

We conducted a retrospective analysis to evaluate the 6-month mortality rate among patients with advanced BTC who received gemcitabine-based chemotherapy between 2012 and 2018 at Chang Gung Memorial Hospital in Linkou. Patients were administered either gemcitabine alone or gemcitabine-based doublet chemotherapy, including GC, GS, and GEMOX, until disease progression or intolerant toxicity was observed. Patients were regularly evaluated through physical examinations, performance status (PS) evaluation, differential blood counts, and serum biochemistry analyses. Imaging studies, such as computed tomography, were performed every 3 months to evaluate the clinical tumor response according to the Response Evaluation Criteria in Solid Tumors (RECIST) guidelines. OS was defined as the time from the first day of chemotherapy until death or the last follow-up date.

### 2.2. Statistical Analysis

Categorical variables were compared using Pearson’s Chi-square test or Fisher’s exact test, based on expected values. Receiver operating characteristic (ROC) curve analysis was used to investigate an optimal cutoff value for the neutrophil to lymphocyte ratio (NLR), monocyte to lymphocyte ratio (MLR), and platelet to lymphocyte ratio (PLR) using the Youden Index. Continuous variables were compared between 2 independent groups using the Mann–Whitney U test. Survival was assessed using the Kaplan–Meier curve, and the log-rank test was applied for comparisons between groups. A Bayesian logistic regression model was constructed to investigate the multivariable relationships between predictors and 6-month mortality to identify independent factors [17]. Cox regression analysis was used for multivariate analyses and to formulate the nomogram.

### 2.3. Nomogram Creation

A nomogram was analyzed by R software (version 2.14.1,R Core Team, 2021, R Foundation for Statistical Computing, Vienna, Austria) with the rms package and other dependent packages (http://www.r-project.org/, accessed on 9 April 2021) [18]. We used categorical variables based on the results of ROC curve analyses. The concordance index (C-index) was applied to measure the performance of the nomogram. A calibration curve was plotted by comparing the nomogram-predicted versus observed probability of survival. For internal validation, bootstrapping with 1000 resamples was used.

### 2.4. Performance of the Nomogram

The performance of the nomogram was analyzed using Nagelkerke’s R squared, which provided the explanation power of the model; the Harrell concordance index (C-statistic), which is used to measure the goodness of fit for binary outcomes in a logistic regression model; and a calibration plot using the Hosmer–Lemeshow goodness of fit test, which plots the nomogram-predicted versus observed probability of survival. To perform an internal validation of the predictive model, we used 1000 bootstrap-sampling repetitions to derive estimates of optimism to reduce the overfit bias.

### 2.5. Statistical Software

A nomogram was analyzed using R software with the rms package and other dependent packages (R Core Team (2021). R: A language and environment for statistical computing. R Foundation for Statistical Computing, Vienna, Austria). SPSS (IBM SPSS Statistics for Windows, Version 20.0. Armonk, NY, USA: IBM Corp.) and SigmaPlot (version 11.0, from Systat Software, Inc., San Jose, CA, USA) were used. The data were fit using a modified 3-parameter exponential decay in SigmaPlot. A *p*-value ≤ 0.05 was considered significant.

## 3. Results

### 3.1. Clinicopathological Characteristics of the Included Patients

**Figure 1:**
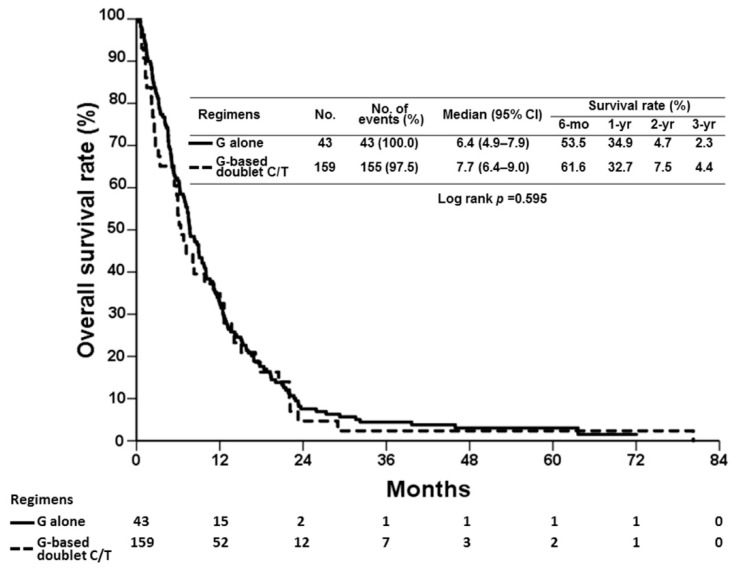
Kaplan–Meier survival curves for overall survival for all patients, stratified according to the chemotherapy (C/T) regimen received: gemcitabine (G) alone or G-based doublet C/T; 95% CI, 95% confidence interval. mo, month; yr, year.

The demographic features of 202 BTC patients who received gemcitabine-based chemotherapy, including 43 patients receiving gemcitabine alone and 159 patients treated with gemcitabine-based chemotherapy in combination with other agents, are shown in Table 1. The study population included 102 men and 100 women with advanced BTC, with a median age of 63 years (range: 31–81 years). Until March 2020, the median OS values were 6.4 and 7.7 months for gemcitabine alone and gemcitabine-based doublet chemotherapy, respectively, which were not significantly different (*p* = 0.595) (Figure 1). The one-year survival rates were 34.9% and 32.7% for gemcitabine alone and gemcitabine-based doublet chemotherapy, respectively.

Most patients (59.4%, *n* = 120) had ICCA, and 85.6% (*n* = 173) of patients were defined as PS ≤ 1. The overall response rate was 11.9%, and 34.2% of patients underwent no response assessment due to a clinically rapid and early deterioration, indicating the aggressive nature of advanced BTC in real-world practice.

### 3.2. Independent Prognostic Factors in the Entire Cohort

Pretreatment characteristics and clinical tumor responses were collected and analyzed by univariate analysis; these are summarized in Table 2. The univariate analysis revealed 10 significant factors, including gender, primary tumor type, PS, NLR, MLR, PLR, albumin, alkaline phosphatase (ALP), liver metastasis, and clinical tumor response (Table 2).

Multivariate analysis was performed using Bayesian logistic regression and eight parameters, including gender, primary tumor type, PS, NLR, MLR, PLR, ALP, and liver metastasis, which identified four independent factors associated with survival: gender, MLR, ALP, and liver metastasis. Clinical tumor response was not included because this was not a pretreatment factor. Although pretreatment albumin was identified as a prognostic factor by univariate analysis, it was excluded from this analysis because more than 10% of patients had missing data.

We further created a nomogram using Cox proportional model analysis with the four parameters identified as independent factors in the multivariate analysis: male sex (hazard ratio (HR): 1.935; 95% confidence interval (CI): 1.001–3.739; *p* = 0.049); MLR ≥ 0.39 (HR: 6.620; 95% CI: 3.421–12.811; *p* ≤ 0.0001); ALP > 94 (HR: 2.322; 95% CI: 0.995–5.421; *p* = 0.051); and the presence of liver metastasis (HR: 2.251; 95% CI: 1.15–4.396; *p* = 0.017; see Table 3).

### 3.3. Prognostic Nomogram for 6-Month Mortality Estimation

**Figure 2:**
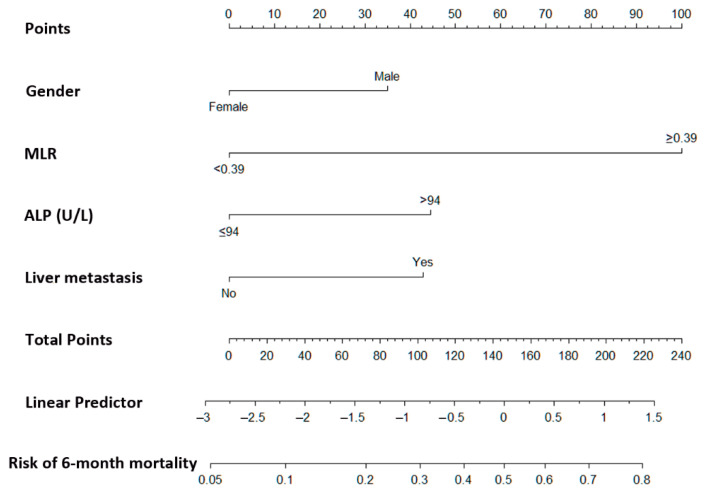
A nomogram was generated to predict the 6-month mortality rate of patients with biliary tract cancer undergoing gemcitabine-based chemotherapy. MLR, monocyte to lymphocyte ratio; ALP, alkaline phosphatase.

A nomogram was constructed based on the results of the final multivariable model (Figure 2). The formula (Table 4) included gender (male: 35 points; female: 0 points), MLR (<0.39: 0 points; ≥0.39: 100 points), ALP (≤94: 0 points; >94: 45 points), and liver metastasis (presence: 43 points; absence: 0 point). A logistic regression model was derived to predict 6-month mortality, ascertained by the sum of all points factored (Table 4 and Figure 2). Higher values correspond to higher 6-month mortality estimates. For example, a patient with all poor prognostic factors (male, MLR ≥ 0.39, ALP > 94, and liver metastasis) would register a score of 223 points, indicating a 6-month mortality estimate of more than 80%.

**Figure 3:**
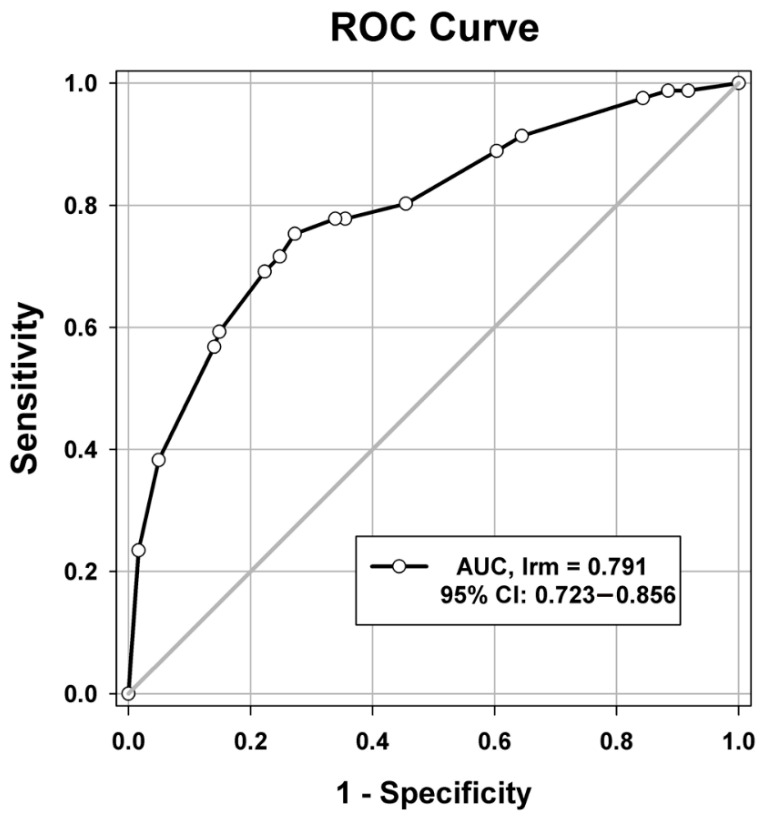
Receiver operating characteristic (ROC) curve analysis was used to evaluate the discrimination ability of the nomogram to predict the 6-month mortality risk. AUC, area under the ROC curve; 95% CI, 95% confidence interval.

**Figure 4:**
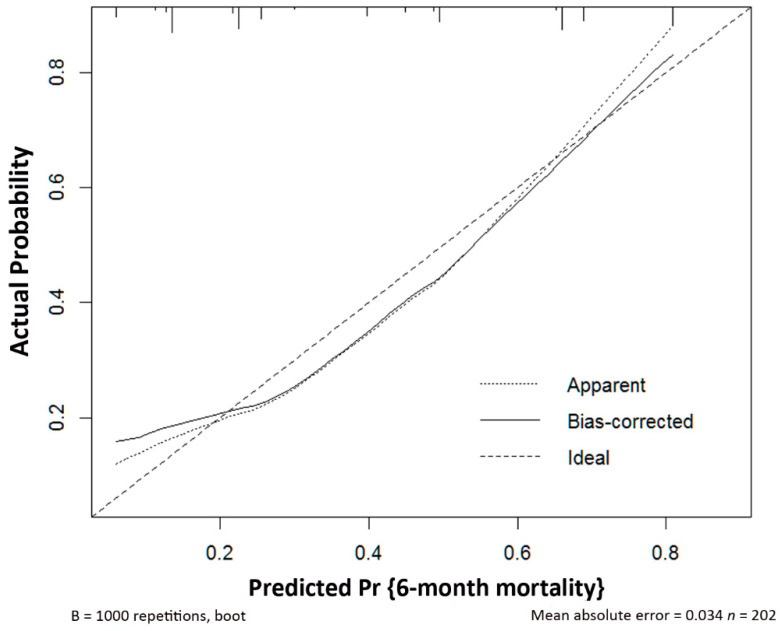
Calibration of the risk prediction model.

**Figure 5:**
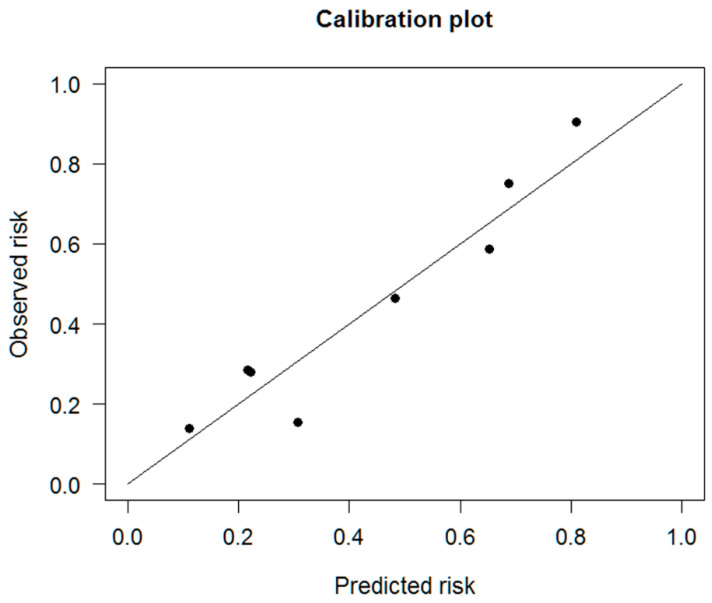
Calibration plot for the Hosmer–Lemeshow goodness of fit test for the risk prediction model.

The performance of this nomogram is summarized in Table 5. A Nagelkerke’s R-squared value of 0.205, which is between 0.13 and 0.25, indicates a medium-sized effect [19]. To evaluate the discrimination and calibration of this nomogram model for the prediction of 6-month mortality [20], Harrell’s concordance index for the model was calculated. The concordance index value of 0.791 indicated the good to strong discrimination ability for this model (Figure 3). In addition, the calibration curve for the probability of survival 6 months after starting gemcitabine-based chemotherapy revealed a good agreement between the nomogram prediction and actual observations (Figure 4). The Hosmer–Lemeshow goodness of fit test was performed to compare the observed and predicted mortality according to the decile of predicted probability. The χ^2^ value was 6.118 (df = 6, *p* = 0.4101), indicating that the model was well calibrated (Figure 5). These parameters indicated that this nomogram represents a good model for the prediction of 6-month mortality.

## 4. Discussion

To the best of our knowledge, this is the first large-cohort study to establish the prediction of the 6-month mortality rate among patients with BTCs using a nomogram. Male sex, MLR ≥ 0.39, ALP > 94, and the presence of liver metastasis were the four independent factors selected for nomogram establishment. The discrimination and calibration analyses for this nomogram model indicated that the nomogram represents a good model for the prediction of 6-month mortality among BTC patients. Therefore, this model could be used in clinical practice to evaluate therapeutic options.

A previous study by Salati et al. [21] established a prognostic model based on the baseline neutrophil count, lymphocyte–monocyte ratio, neutrophil–lymphocyte ratio, and albumin (A.L.A.N.) obtained from an exploratory cohort of 123 BTC patients and validated their model using another cohort of 60 patients. CEA, PS, and disease status (locally advanced vs. metastatic disease) were identified as independent prognostic factors, but they were not included in the A.L.A.N. model. In contrast, we identified MLR, gender, ALP, and liver metastasis as independent prognostic factors, which represent broader factors than those included in the A.L.A.N. model. Lymphocytes are considered to represent the anti-tumor immunological reaction and contribute to a good prognosis, whereas monocytes are associated with proinflammatory cytokines and a poor prognosis. MLR, a simple and easily assessed biomarker that reflects the anti-tumor immunity balance, was identified as a prognostic factor in both studies, indicating its importance in BTC patients [22]. In addition, this study was performed in Taiwan, resulting in the development of a model that may be more suitable for the Asian population than the A.L.A.N. model based on European patients, as it is possible different etiologies, risk factors, and genetics causes BTCs to affect Asian and European populations differently. Furthermore, we used the 6-month mortality rate as a specific and simple endpoint, rather than categorizing patients into risk groups. Although the A.L.A.N. scores divided the patients into high-, intermittent-, and low-risk groups, this score did not accurately predict survival, as the exploratory and validation cohorts showed distinct survival outcomes (median OS: 22 months for the low-risk group in the exploratory cohort and 13 months for the low-risk group in the validation cohort).

In our previous study [15], clinical tumor response was identified as the most significant prognostic factor for BTC patients; however, the tumor response is uncertain prior to treatment. Thechallenge for clinicians is to identify those patients who may or may not benefit from specific types of chemotherapy. The 6-month mortality rate might represent a useful surrogate biomarker for the prediction of clinical outcomes. Chemotherapy using gemcitabine-based regimens is optimal for patients with a low 6-month mortality risk, but might not be sufficiently aggressive in patients with a high 6-month mortality risk. Whether clinicians provide more aggressive treatment or palliative supportive care to patients with a high 6-month mortality risk often depends on the patients’ preferences, and a nomogram able to identify the 6-month mortality risk can help clinicians and patients make these decisions. BTC patients who are aware of their 6-month mortality risk when treated with conventional chemotherapy can determine their willingness to undergo such treatments, pursue more aggressive treatments, or accept palliative care.

To improve the OS associated with the limited efficacy of current standard treatments, more effective treatments for both front-line and late-line use must be developed. Although advances in next-generation sequencing, targeted therapies, and immunotherapies have improved overall survival rates in most cancers, the treatments available for advanced BTC remain disappointing. Therefore, the development of next-generation treatments for BTC patients remains necessary [23]. The randomized, open-label, Phase III ABC-06 clinical trial examined oxaliplatin and 5-fluorouracil/leucovorin (FOFLOX) treatment in 162 patients, which indicated improved the OS (6.2 vs. 5.3 months; HR: 0.69, *p* = 0.031) compared to active symptom control in patients who progressed after first-line GC treatment [24]. In addition, only a small and specific population of BTC patients who harbored driver mutations, including FGFR2 fusion [25], IDH1 [26], and BRAF mutations [27], as well as agnostic markers of MSI-H/dMMR status [28] and NRTK fusion [29] are able to benefit from targeted therapies or immunotherapies [23].

In the current study, we included all patients undergoing gemcitabine-based chemotherapy, either as a monotherapy or in combination with other cytotoxic agents. Although GC demonstrated a better OS than gemcitabine alone, no significant difference between these two regimens was found in our real-world experience. The baseline characteristics between patients undergoing gemcitabine monotherapy and gemcitabine-based doublet chemotherapy are compared in Table S1. The patients undergoing gemcitabine monotherapy were older; had higher bilirubin, ALP, and CA199 levels; were more likely to be classified as PS ≥ 2; had albumin < 3.5; and were stented but less likely to present with lung metastasis and ICCA than patients treated with gemcitabine-based doublet chemotherapy. The similar OS observed between these groups might result from the imbalance of these important prognostic factors. As this was not a controlled model, the lack of differences in OS identified among different chemotherapy regimens cannot be used to infer that the efficacies of these chemotherapy regimens are similar. 

The present study had some limitations, including its retrospective nature, which always involves some degree of bias. However, the study established a nomogram based on real-world experience, which may be more optimal for clinicians and patients than data obtained from clinical trials. In addition, a multivariate analysis was performed to adjust for possible confounding factors. The analyzed patients were treated at a high-volume, tertiary-care, single institute with reliable and consistent quality. Second, this study failed to assess the influence of nutritional status, which can be performed using screening tools such as the Malnutrition Universal Screening Tool (MUST) [30] or the Nutritional Risk Screening-2002 (NRS-2002) [31,32]. Due to the retrospective nature of this study, documented assessments of nutritional status were lacking for most patients. Serum albumin and lymphocytes were evaluated in the current study as proxies for nutritional status [33]. Third, this study lacked any external validation cohort due to the rarity of this disease. As an alternative method, we used bootstrapping to strengthen the model, although this is a less optimal approach than external validation.

## 5. Conclusions

In conclusion, we established a nomogram based on four independent pretreatment factors—gender, MLR, ALP, and liver metastasis—using a logistic regression model to predict the 6-month mortality risk, thus providing clinicians and patients with additional information regarding survival outcomes. Future studies are warranted in order to validate this nomogram.

## Figures and Tables

**Table 1:** Patient demographics and clinicopathological features (*n* = 202).

Characteristic	N (%) or Median (IQR)
Age (years)	63.0 (13.0)
Gender	
Male	102 (50.5)
Female	100 (49.5)
ICD-10 cancer site	
C22.1-ICCA	120 (59.4)
C23/C24.9-GB/others	37 (18.36)
C24.0-ECCA	38 (18.8)
C24.1-Ampullary	7 (3.5)
Performance score	
0	31 (15.3)
1	142 (70.3)
2	25 (12.4)
3	4 (2.0)
NLR	3.9 (4.0)
MLR	0.4 (0.3)
PLR	157.4 (119.4)
Albumin (g/dL)	3.7 (0.9)
ALT (U/L)	31.0 (41.0)
Bilirubin (mg/dL)	0.8 (1.0)
ALP (U/L)	169.0 (170.8)
Creatinine (mg/dL)	0.6 (0.4)
CA199 (U/mL)	260.7 (1384.6)
CEA (ng/mL)	3.0 (9.7)
Biliary drainage	
None	132 (65.3)
Internal stenting	13 (6.4)
PTCD	51 (25.2)
Both	6 (3.0)
Tumor involvement	
Primary tumor	
No	15 (7.4)
Yes	187 (92.6)
Regional lymphadenopathy	
No	74 (36.6)
Yes	128 (63.4)
Lung	
No	170 (84.2)
Yes	32 (15.8)
Bone	
No	186 (92.1)
Yes	16 (7.9)
Liver	
No	120 (59.4)
Yes	82 (40.6)
Peritoneum	
No	168 (83.2)
Yes	34 (16.8)
Distant lymphadenopathy	
No	175 (86.6)
Yes	27 (13.4)
Response	
CR/PR	24 (11.9)
SD	25 (12.4)
PD	84 (41.6)
NA	69 (34.2)

IQR, interquartile range; SD, standard deviation; CR, complete response; PR, partial response; SD, stable disease; PD, progressive disease; NA, not assessed; ALP, alkaline phosphatase; ALT, alanine aminotransferase; NLR, neutrophil to lymphocyte ratio; MLR, monocyte to lymphocyte ratio; PLR, platelet to lymphocyte ratio; PTCD, percutaneous transhepatic cholangiography drainage; ICCA, intrahepatic cholangiocarcinoma; ECCA, extrahepatic cholangiocarcinoma; GB, gallbladder; CEA, carcinoembryonic antigen.

**Table 2:** Univariate and multivariate analyses ** of patients treated with G-based chemotherapy.

Predictor Variables	6-Month Mortality		*p*-Value	Odds Ratio	95% CI of Odds Ratio	*p*-Value
	Yes (*n* = 81)	No (*n* = 121)				
Age (years)						
≤65	47 (58.0)	72 (59.5)	0.834			
>65	34 (42.0)	49 (40.5)				
Gender			0.009			
Male	50 (61.7)	52 (43.0)		2.40	1.21–4.76	0.012
Female	31 (38.3)	69 (57.0)		1		
ICD-10 cancer site			0.012			
C22.1-ICCA	51 (63.0)	69 (57.0)		1		
C23/C24.9-GB/others	20 (24.7)	17 (14.0)		2.35	0.97–5.70	0.058
C24.0-ECCA	10 (12.3)	28 (23.1)		0.72	0.29–1.80	0.479
C24.1-Ampullary	0	7 (5.8)		0.07	0.01–1.40	0.081
Performance score			0.009			
0/1	63 (77.8)	110 (90.9)		1		
2/3	18 (22.2)	11 (9.1)		2.28	0.89–5.85	0.087
NLR			<0.0001			
<3.95	24 (29.6)	80 (66.1)		1		
≥3.95	57 (70.4)	41 (33.9)		1.27	0.49–3.30	0.619
MLR			<0.0001			
<0.39	20 (24.7)	86 (71.1)		1		
≥0.39	61 (75.3)	35 (28.9)		4.50	1.74–11.69	0.002
PLR			0.005			
<147.2	24 (29.6)	60 (49.6)		1		
≥147.2	57 (70.4)	61 (50.4)		0.99	0.45–2.19	0.975
Albumin (g/dL) *			0.002			
<3.5	38 (51.4)	30 (28.3)				
≥3.5	36 (48.6)	76 (71.7)				
ALT (U/L)			0.060			
≤36	54 (66.7)	64 (53.3)				
>36	27 (33.3)	56 (46.7)				
Bilirubin (mg/dL)			0.209			
≤1.3	54 (67.5)	90 (75.6)				
>1.3	26 (32.5)	29 (24.4)				
ALP (U/L)			0.021			
≤94	11 (13.6)	33 (27.3)		1		
>94	70 (86.4)	88 (72.7)		2.65	1.11–6.33	0.029
Creatinine (mg/dL)			0.062			
≤1.27	75 (92.6)	119 (98.3)				
>1.27	6 (7.4)	2 (1.7)				
CA199 (U/mL)			0.858			
<37	21 (26.6)	29 (25.4)				
≥37	58 (73.4)	85 (74.6)				
CEA (ng/mL)			0.638			
≤5	49 (60.5)	74 (63.8)				
>5	32 (39.5)	42 (36.2)				
Biliary drainage			0.589			
None	51 (63.0)	81 (66.9)				
Internal stenting	5 (6.2)	8 (6.6)				
PTCD	21 (25.9)	30 (24.8)				
Both	4 (4.9)	2 (1.7)				
Tumor involvement						
Primary tumor			0.099			
No	3 (3.7)	12 (9.9)				
Yes	78 (96.3)	109 (90.1)				
Regional lymphadenopathy			0.841			
No	29 (35.8)	45 (37.2)				
Yes	52 (64.2)	76 (62.8)				
Lung			0.394			
No	66 (81.5)	104 (86.0)				
Yes	15 (18.5)	17 (14.0)				
Bone			0.170			
No	72 (88.9)	114 (94.2)				
Yes	9 (11.1)	7 (5.8)				
Liver			0.008			
No	39 (48.1)	81 (66.9)		1		
Yes	42 (51.9)	40 (33.1)		2.38	1.17–4.83	0.017
Peritoneum			0.094			
No	63 (77.8)	105 (86.8)				
Yes	18 (22.2)	16 (13.2)				
Distant lymphadenopathy			0.621			
No	69 (85.2)	106 (87.6)				
Yes	12 (14.8)	15 (12.4)				
Regimen			0.334			
G alone	20 (24.7)	23 (19.0)				
G-based doublet C/T	61 (75.3)	98 (81.0)				
Response			<0.0001			
CR/RR	2 (2.5)	22 (18.2)				
SD	9 (11.1)	60 (49.6)				
PD	46 (56.8)	38 (31.4)				
NA	24 (29.6)	1 (0.8)				

Figures are numbers with percentages in parentheses, unless otherwise stated. ICCA, intrahepatic cholangiocarcinoma; CR, complete response; PR, partial response; SD, stable disease; PD, progressive disease; NA, not assessed; ALP, alkaline phosphatase; ALT, alanine aminotransferase; NLR, neutrophil to lymphocyte ratio; MLR, monocyte to lymphocyte ratio; PLR, platelet to lymphocyte ratio; PTCD, percutaneous transhepatic cholangiography drainage; G, gemcitabine; C/T, chemotherapy; ECCA, extrahepatic cholangiocarcinoma; GB, gallbladder; 95% CI, 95% confidence interval. * Variables with large numbers of missing values were omitted from the multivariate analysis. ** Multivariate analysis using *Bayesian logistic regression to* solve the problem of separation in logistic regression.

**Table 3:** Multivariable model of predictors for 6-month mortality risk among patients treated with G-based chemotherapy.

Predictor Variables	Hazard Ratio (HR)	95% CI for HR		*p*-Value
		Lower	Upper	
Gender				
Male	1.935	1.001	3.739	0.049
Female	Reference			
MLR				
<0.39	Reference			
≥0.39	6.620	3.421	12.811	<0.0001
ALP (U/L)				
≤94	Reference			
>94	2.322	0.995	5.421	0.051
Liver metastasis				
No	Reference			
Yes	2.251	1.153	4.396	0.017

CI, Confidence interval; HR, hazard ratio; MLR, monocyte to lymphocyte ratio; ALP, alkaline phosphatase. Multivariate analysis using *logistic regression.*

**Table 4:** Point-based scoring system for the predictor variables and the probability of 6-month mortality associated with different total point values.

Predictor Variables	Points Assigned
Gender	
Male	35
Female	0
MLR	
<0.39	0
≥0.39	100
ALP (U/L)	
≤94	0
>94	45
Liver metastasis	
No	0
Yes	43
Total score	Probability of 6-month mortality (%)
30	0.10
73	0.20
101	0.30
125	0.40
146	0.50
167	0.60
191	0.70
219	0.80

**Table 5:** Performance measures using logistic regression for the prediction of the 6-month mortality risk.

Model	Overall Performance Measure	Discrimination	Calibration
	Nagelkerke R^2^	C Statistic	Hosmer–Lemeshow Test
Risk prediction model	0.205	0.791	0.410

## Data Availability

The data presented in this study are available on request from the corresponding author. The data are not publicly available due to ethical regulations.

## Supplementary Materials

The following are available online at https://www.mdpi.com/article/10.3390/cancers13133139/s1, Table S1: Characteristics of patients stratified by chemotherapy regimens.
