# Supplementary material for: Establishment of a Pretreatment Nomogram to Predict the 6-Month Mortality Rate of Patients with Advanced Biliary Tract Cancers Undergoing Gemcitabine-Based Chemotherapy
Source: Cancers (Basel). 2021 Jun 23;13(13):3139. doi: 10.3390/cancers13133139 (PMC8268608; doi:10.3390/cancers13133139)
Supplement: Supplementary file 1 [file cancers-13-03139-s001.zip › cancers-1245576-supplementary.pdf]

cancers MDPI

# Supplementary Material: Establishment of a Pretreatment Nomogram to Predict the 6-Month Mortality Rate of Patients with Advanced Biliary Tract Cancers Undergoing Gemcitabine-based Chemotherapy

**Chiao-En Wu, Wen-Kuan Huang, Wen-Chi Chou, Chia-Hsun Hsieh, John Wen-Cheng Chang, Cheng-Yu Lin, Chun-Nan Yeh and Jen-Shi Chen**

**Table S1.** Characteristics of patients stratified by chemotherapy regimens.

| Characteristic | G alone ($n$ = 43) | G-based double C/T ($n$ = 159) | $p$-value |
|---|---|---|---|
| Age (years), median (IQR) | 66.0 (15.0) | 62.0 (14.0) | 0.023 |
| ≤65 | 20 (46.5) | 99 (62.3) | 0.063 |
| >65 | 23 (53.5) | 60 (37.7) | |
| Gender | | | 0.351 |
| Male | 19 (44.2) | 83 (52.2) | |
| Female | 24 (55.8) | 76 (47.8) | |
| ICD-10 cancer site | | | <0.001 |
| C22.1-ICCA | 14 (32.6) | 106 (66.7) | |
| C23/C24.9-GB/others | 10 (23.3) | 27 (17.0) | |
| C24.0-ECCA | 17 (39.5) | 21 (13.2) | |
| C24.1-Ampullary | 2 (4.7) | 5 (3.1) | |
| Performance score | | | 0.004 |
| 0/1 | 31 (72.1) | 142 (89.3) | |
| 2/3 | 12 (27.9) | 17 (10.7) | |
| NLR, median (IQR) | 4.06 (5.03) | 3.88 (3.56) | 0.510 |
| <3.95 | 21 (48.8) | 83 (52.2) | 0.695 |
| ≥3.95 | 22 (51.2) | 76 (47.8) | |
| MLR, median (IQR) | 0.35 (0.40) | 0.37 0.31) | 0.541 |
| <0.39 | 24 (55.8) | 82 (51.6) | 0.621 |
| ≥0.39 | 19 (44.2) | 77 (48.4) | |
| PLR, median (IQR) | 184.37 (123.95) | 152.60 (121.94) | 0.106 |
| <147.2 | 14 (32.6) | 70 (44.0) | 0.176 |
| ≥147.2 | 29 (67.4) | 89 (56.0) | |
| Albumin (g/dL), median (IQR) | 3.34 (0.90) | 3.80 (0.92) | 0.106 |
| <3.5 | 23 (59.0) | 45 (31.9) | 0.002 |
| ≥3.5 | 16 (41.0) | 96 (68.1) | |
| ALT (U/L), median (IQR) | 37.0 (46.0) | 30.0 (35.3) | 0.348 |
| ≤36 | 21 (48.8) | 97 (61.4) | 0.138 |
| >36 | 22 (51.2) | 61 (38.6) | |
| Bilirubin (mg/dL), median (IQR) | 1.15 (1.10) | 0.70 (0.70) | 0.002 |
| ≤1.3 | 23 (54.8) | 121 (77.1) | 0.004 |
| >1.3 | 19 (45.2) | 36 (22.9) | |
| ALP (U/L), median (IQR) | 255.00 (255.00) | 159.00 (153.00) | 0.008 |
| ≤94 | 6 (14.0) | 38 (23.9) | 0.161 |
| >94 | 37 (86.0) | 121 (76.1) | |
| Creatinine (mg/dL), median (IQR) | 0.62 (0.48) | 0.65 (0.32) | 0.591 |
| ≤1.27 | 40 (93.0) | 154 (96.9) | 0.371 |

| | | | |
|---|---|---|---|
| >1.27 | 3 (7.0) | 5 (3.1) | |
| CA199 (U/mL), median (IQR) | 550.80 (960.10) | 165.0 (1686.43) | 0.038 |
| <37 | 3 (8.1) | 47 (30.1) | 0.006 |
| ≥37 | 34 (91.9) | 109 (69.9) | |
| CEA (ng/mL), median (IQR) | 2.45 (13.64) | 3.10 (8.58) | 0.821 |
| ≤5 | 24 (61.5) | 99 (62.7) | 0.897 |
| >5 | 15 (38.5) | 59 (37.3) | |
| Biliary drainage | | | 0.014 |
| None | 21 (48.8) | 111 (69.8) | |
| Internal stenting | 4 (9.3) | 9 (5.7) | |
| PTCD | 18 (41.9) | 33 (20.8) | |
| Both | 0 | 6 (3.8) | |
| Tumor involvement | | | |
| Primary tumor | | | >0.999 |
| No | 3 (7.0) | 12 (7.5) | |
| Yes | 40 (93.0) | 147 (92.5) | |
| Regional lymphadenopathy | | | 0.930 |
| No | 16 (37.2) | 58 (36.5) | |
| Yes | 27 (62.8) | 101 (63.5) | |
| Lung | | | 0.023 |
| No | 41 (95.3) | 129 (81.1) | |
| Yes | 2 (4.7) | 30 (18.9) | |
| Bone | | | 0.201 |
| No | 42 (97.7) | 144 (90.6) | |
| Yes | 1 (2.3) | 15 (9.4) | |
| Liver | | | 0.390 |
| No | 28 (65.1) | 92 (57.9) | |
| Yes | 15 (34.9) | 67 (42.1) | |
| Peritoneum | | | 0.304 |
| No | 38 (88.4) | 130 (81.8) | |
| Yes | 5 (11.6) | 29 (18.2) | |
| Distant lymphadenopathy | | | 0.377 |
| No | 39 (90.7) | 136 (85.5) | |
| Yes | 4 (9.3) | 23 (14.5) | |
| Response | | | 0.468 |
| CR/RR | 6 (14.0) | 18 (11.3) | |
| SD | 8 (18.6) | 17 (10.7) | |
| PD | 15 (34.9) | 69 (43.4) | |
| NA | 14 (32.6) | 55 (34.6) | |

IQR, interquartile range, CR, complete response; PR, partial response; SD, stable disease, PD, progressive disease, NA, not assessed; ALP, alkaline phosphatase; ALT, alanine aminotransferase; NLR, neutrophil to lymphocyte ratio; MLR, monocyte to lymphocyte ratio; PLR, platelet to lymphocyte ratio, LAP, Lymphadenopathy, PTCD, percutaneous transhepatic cholangiography drainage; ICCA, intrahepatic cholangiocarcinoma; ECCA, extrahepatic cholangiocarcinoma; GB: gallbladder; CEA, carcinoembryonic antigen.
